# Curriculum Innovation: A Master Clinician Residency Track in Neurology

**DOI:** 10.1212/NE9.0000000000200302

**Published:** 2026-03-23

**Authors:** Megan Richie, Vanja C. Douglas, S. Andrew Josephson, Gurpreet Dhaliwal

**Affiliations:** 1 Neurology, University of California San Francisco;; 2 Medicine, University of California San Francisco; and; 3 Medical Service, San Francisco VA Medical Center, CA.

## Abstract

**Background and Objectives:**

While academic centers have traditionally valued faculty research and educational accomplishments, clinical excellence is often assumed or underappreciated. Accordingly, neurology residency programs frequently offer structured pathways for aspiring clinician-scientists or educators, but few offer dedicated training to launch residents into academic careers focused on clinical mastery. Guided by self-regulated learning principles and the Master Adaptive Learner framework, the aim of this curriculum was to equip neurology postgraduate year (PGY)-4 residents with the foundational skills of the master clinician by advancing the following: (1) clinical acumen and diagnostic reasoning; (2) a scholarly and evidence-informed approach to their work; (3) outstanding communication, professionalism, and humanism; and (4) effective navigation and leadership within complex health care systems.

**Methods:**

Using the Kern six-step curriculum design framework, we developed a 6-month Master Clinician track that included didactics, customized clinical rotations, reflective patient portfolios, and a scholarly project. Didactics addressed topics such as cognitive bias, adaptive expertise, communication, and clinical uncertainty. Clinical rotations were learning-focused experiences tailored to resident interests. Track residents maintained 2 patient portfolios: one for in-depth case analysis (“depth” portfolio) and another to track diagnostic accuracy (“breadth” portfolio). The scholarly project reinforced a reflective approach to patient care and promoted academic inquiry skills. Assessments of the track included an exit survey on completion, patient portfolio audits, and post-track surveys for 3 years after graduation from residency.

**Results:**

Five residents completed the track in the first 5 years (2017–2022). Baseline residency milestone ratings were similar between participants and nonparticipants. Track components received high ratings (≥4.6 on a 5-point scale), with didactics on cognitive bias and teaching skills rated highest. Portfolios averaged 32 cases (breadth) and 8 cases (depth). Residents highlighted clinical uncertainty, communication, and cognitive bias as the most relevant sessions for patient care. Over 3 years of follow-up, high satisfaction with the track persisted and most enrollees elected academic clinical or clinician-educator careers.

**Discussion:**

A residency track that used didactics, customized clinical experiences, case-tracking, and reflective practices to foster clinical excellence was well received by residents. Neurology programs should consider similar tracks to develop academic clinicians focused on excellence in patient care.

## Introduction

Clinical expertise is rarely promoted as a career goal in academic medicine, where clinical work is often undervalued or inadequately supported.^1-3^ A study of career success among academic neurologists used publication records as the primary indicator without assessing clinical expertise.^4^ This lack of recognition has been linked to physician attrition from academia.^5^ Compared with their peers in research or education, clinically oriented faculty at academic medical centers experience lower promotion rates and less career satisfaction.^3,5-7^ The physician-scientist and clinician-educator are well-recognized career pathways in neurology, but there is no established professional identity for academic faculty whose primary focus is clinical care.^8^

This relative undervaluing of clinical expertise is at odds with the growing need for highly skilled clinicians in academic medical centers. Such clinicians are essential in the selection of sophisticated testing and treatments and coordination of extended medical interprofessional teams that account for improved patient outcomes in teaching hospitals, particularly for complex patients. Academic medical centers have been associated with a small but meaningful reduction in 30-day postadmission mortality, and the degree of that benefit rose as patient complexity increased.^9^ Furthermore, many academic medical centers are seeking to expand their clinical footprint.^10^ These factors create a pressing need for master clinicians capable of caring for these complex patients in an academic environment.^11^ Outstanding clinicians also support broader missions of academics, including disseminating excellence through modeling, teaching, and mentorship of learners and informing investigators with their clinical insights, rendering the professional skillset required of a master clinician within an academic environment distinct from that of highly skilled clinicians in the community.

While many neurology training programs have specialized tracks to prepare residents for research and education careers, few provide pathways for residents whose primary career goal is to become outstanding clinicians at academic medical centers.^12,13^ This gap has been recognized by some recently developed fellowships focusing on training expert general neurologists who are skilled at managing uncertainty and caring for patients with complex neurologic symptoms.^14^ Residents aspiring to become master clinicians within academic neurology may benefit from specialized training that goes beyond the fundamental clinical topics taught in residency programs to promote skills focused on development of clinical mastery in the face of complexity and uncertainty.

To meet this emerging need, we developed a voluntary 6-month track, hereafter the Master Clinician track, to introduce neurology PGY-4 residents with the skills to become academic neurologists, with a primary career focus on clinical excellence. Our curriculum was designed to promote self-regulated learning (SRL) skills within the Master Adaptive Learner (MAL) framework.^15,16^

### Objectives

The primary objective of the track was to provide experiences that introduced a select group of neurology residents in their final year of residency to the skills necessary to become academic neurologists with a career focused on clinical excellence. Four traits central to the multidimensional construct of the master clinician informed the development of specific supporting objectives (Table 1):

1. Advanced clinical reasoning:
  1. Develop systematic approaches to complex clinical situations.
  2. Describe factors that contribute to medical errors.
  3. Appreciate multidisciplinary perspectives on neurologic illnesses.
  4. Forecast and revisit diagnostic accuracy.
2. Scholarly approach to work:
  1. Consistently use medical evidence.
  2. Investigate a clinically relevant question in depth.
3. Outstanding communication skills, professionalism, and humanism:
  1. Identify factors contributing to discrimination and/or challenges in communication and professionalism in patient care.
4. Skillful navigation of the health care system:
  1. Appreciate the roles that different health care disciplines play in neurologic care.
  2. Consider cost/benefit analyses as they relate to patient cases.

**Table 1:** Master Clinician Track Supporting Objectives and Educational Strategies/Implementation

Master clinician trait	Supporting objective	Educational strategy	Assessment
Advanced clinical acumen and clinical reasoning	Develop systematic approaches to complex clinical situations	• “Adaptive expertise and deliberate practice” didactic	• “Depth” case portfolio • In-depth case analyses
		• 2–6 wk of learning-focused clinical rotations in neurology	
	Describe factors that contribute to medical errors	• “Decision making and cognitive biases” didactic	
	Appreciate multidisciplinary perspectives on neurologic illnesses	• 2–6 wk of non-neurologic multidisciplinary clinical rotations	
	Forecast and revisit diagnostic accuracy	• “Adaptive expertise and deliberate practice” didactic	• “Breadth” case portfolio
Scholarly approach to work	Consistently use medical evidence	• 2–6 wk of learning-focused clinical rotations in neurology	• “Depth” case portfolio • Project completion
	Investigate a clinically relevant question in depth	• Scholarly project	
Outstanding communication skills, professionalism, and humanism	Identify factors contributing to discrimination and/or challenges in communication and professionalism in patient care	• “Enhancing relationship-centered communication skills” half-day course • “Professionalism and physicianship” didactic • “Cultural humility” didactic	• “Depth” case portfolio • In-depth case analysis
Skillful navigation of the health care system	Appreciate the roles that different health care disciplines play in neurologic care	• 2–6 wk of non-neurologic multidisciplinary clinical rotations	• “Depth” case portfolio
	Consider cost/benefit analyses as they relate to patient cases	• “Cost/benefit analysis” didactic	

## Methods

The development of the Master Clinician track was guided by the Kern curriculum design framework. To assess the need for a residency track supporting clinical excellence, we surveyed members of an academic clinical neurology department (N = 56, including 18 residents, 1 fellow, and 37 faculty members). The needs assessment survey is given in eAppendix 1. Respondents rated departmental support for basic science higher than for clinical work (4.4 vs 4.0 out of 5, *p* = 0.02) and were more likely to recommend basic science as a career (4.2 vs 3.8, *p* = 0.008). Respondents perceived clinical research as a more viable career pathway than clinical neurology (3.4 vs 3.1, *p* = 0.01), and they were more likely to recommend a research career than a clinical career (4.4 vs 3.8, *p* = 0.004). These trends persisted or were amplified in analyses limited to faculty respondents.

### Conceptual Foundations

Pursuing our goal of positioning neurology residents on a trajectory to become expert clinicians required an understanding of how a master clinician is defined and the process of becoming one. Multiple clinical excellence studies, primarily in academic medical centers, have converged on a multidimensional construct of the master clinician as a physician with advanced clinical acumen; enthusiasm for patient care, mentorship, and teaching; exceptional communication, professionalism, and humanism skills; a scholarly approach to work; and skillful navigation of the health care system.^17-20^ While this profile provides a goal for aspiring trainees, less is known about how residency programs can orient learners toward this goal.

Becoming an expert in any field requires sustained, goal-oriented activities to continually develop skills.^16,21-23^ Theories of expertise such as deliberate practice specify requisite components of this process including focused and repeated practice, immediate feedback, progressive challenge, and long-term commitment to continual improvement. However, medical training programs and practice settings are designed to ensure acceptable medical competence; they are not designed with the specific goal of each member operating at advanced levels with an upward trajectory across the multitude of skills that characterize master clinicians. As a result, clinical excellence often becomes an individual pursuit, with trainees relying on SRL to transcend competency standards. SRL involves setting goals, monitoring performance, and reflecting on outcomes to guide future improvement.^16,19,22-25^ Providing residents with tools to facilitate SRL was, therefore, a fundamental aspect of the Master Clinician track.

To care for the complex patients common to academic medicine, a master clinician must be able to function effectively in situations of clinical uncertainty and continually learn and adapt their methods when routine approaches fail. We used a MAL framework to guide the development of such adaptive expertise. The MAL model is driven by SRL and organized into 4 general phases: planning, in which learners identify a gap in their practice and set goals and a plan to address the gap; learning, in which learners execute their plan; assessing, when learners try out their new knowledge or skills, grow in comfort with their use, and assess their effectiveness; and adjusting, when learners incorporate their new knowledge and skills into their daily routines and existing systems. Specific didactics, therefore, were designed to equip residents with skills and tools integral to the MAL model, including identifying sources of uncertainty, new resources to incorporate, and self-assessment.

Finally, achieving our goal of supporting academic clinical neurology as a career pathway for residents required examining how the skills and knowledge of a master clinician differ in an academic setting compared with those in nonacademic institutions. Academic master clinicians are often distinguished by their excellence in teaching and mentorship, navigation of complex health care institutions, and scholarly approach to clinical practice.^18,19,25^ Our training program, therefore, contained curricular elements that covered these topics, including both didactics and a scholarly project.

### Educational Strategies

Lectures and group discussion–based didactics were selected to introduce concepts and strategies needed to set learning goals, monitor progress, and seek new challenges in the pursuit of clinical expertise. Focused clinical experiences, patient portfolios, and an independent study project allowed residents to put these concepts into practice and develop foundational habits. These educational strategies were aligned with SRL and the MAL framework, which outlines a process of planning, learning, assessing, and adjusting to routine and novel challenges.^15^ The educational strategies were mapped to the Accreditation Council for Graduate Medical Education core clinical competencies to maintain alignment with the current residency training framework.

### Implementation

The 6-month Master Clinician track included 2–3 months of clinical rotations and 2–4 months for a scholarly project and the longitudinal didactic program. Residents applied to the track by submitting a proposal for their scholarly project. Proposals were reviewed and refined by a committee of faculty members, who then voted to determine resident acceptance in the track. This track complemented several other flexible residency options already available to neurology residents at our institution, allowing them to dedicate 6 months of their final year of training to academic areas such as neuroscience research, clinical research, global health, or education.^26^ The flexible residency program was funded by a philanthropic endowment.

During the track, clinical experiences were scheduled in continuous blocks of time (inpatient setting) or interspersed within research time (outpatient clinics). Didactics were led by faculty members; attendance was capped at 6 residents (track and nontrack) to promote discussion and interaction. The core neurology residency curriculum already covered clinical neurology in depth; the track curriculum didactics, therefore, focused on metacognitive skills and systems-based practice skills that are foundational to clinical excellence (Table 1). Topics included cognitive biases, adaptive expertise, clinical uncertainty, deliberate practice, cultural humility, communication strategies, professionalism, longitudinal patient follow-up with self-directed review, cost-benefit analysis, teaching skills, and optimizing continuing medical education (CME) and maintenance of certification. Each session was 1 hour, with 30–45 minutes of lecture followed by group discussion. Faculty didactic leaders were volunteers recruited from the departments of neurology, pediatrics, and internal medicine, based on their knowledge and experience with each topic.

The didactic curriculum also included 3 in-depth case analyses designed to promote reflective practice and improve diagnostic skills.^27^ These cases were spread out over the final year of residency and became progressively more challenging. In these didactics, a faculty member presented a complex unknown case to residents and asked them to perform an independent written analysis. The faculty member then led a collective debrief, provided case closure, and summarized salient teaching points.

All track participants completed 2–3 months of clinical rotations aligned with their interests, including at least 2 weeks in non-neurologic settings (e.g., palliative care, rehabilitation medicine, neurosurgery, or primary care) to broaden their exposure to related disciplines and strengthen their systems navigation skills. Each resident also spent at least 2 weeks serving as an additional member of a neurology inpatient team, allowing them to assume care of a lower volume of patients than they would have as core members on those inpatient rotations. This learning-focused structure mirrored other patient-volume reduction initiatives, which give residents time to apply newly acquired concepts and skills.^28^

**Figure 1:**
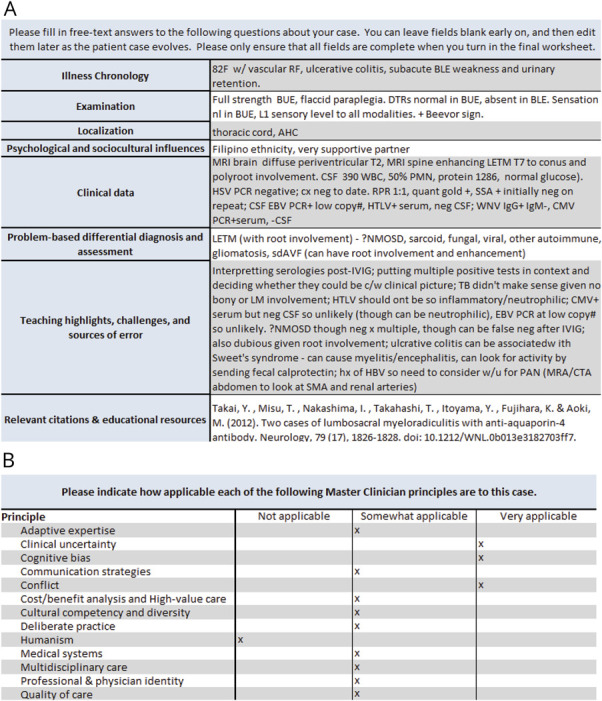
Example Depth Portfolio (A) Track residents recorded in-depth analysis of selected cases from their learning-focused clinical rotations. (B) Residents rated the relevance of track curriculum topics for each individual case.

Track participants maintained 2 clinical case portfolios, which they reviewed with a faculty member at the completion of their residency. The first (“depth” portfolio, Figure 1) focused on in-depth analysis of selected cases from their learning-focused clinical rotations. Residents rated the relevance of track curriculum topics for each individual case. This portfolio mirrored the format of the 3 in-depth case analyses included in the track didactics, aiming to promote a structured approach to self-directed learning and to reinforce didactic session concepts.^29^

**Figure 2:**
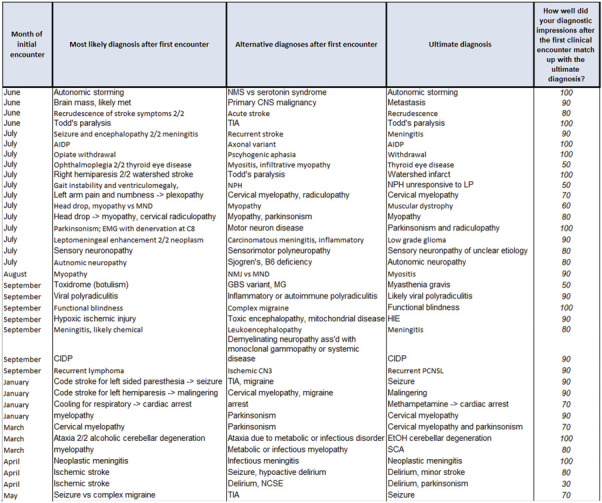
Example Breadth Portfolio Track residents recorded all inpatient and outpatient cases they personally evaluated during the track, indicating their top suspected diagnoses. After track completion, residents performed chart review and recorded their subjective diagnostic accuracy.

The second case portfolio (“breadth” portfolio, Figure 2) focused on diagnostic accuracy. Residents recorded each inpatient and outpatient case they evaluated during the track along with their top 1–3 suspected diagnoses. At track completion between 2 and 10 months later, they performed chart review and examined how well their original diagnostic impressions matched up with the patient's ultimate diagnosis. This process prompted reflection and encouraged the habit of seeking feedback to continuously refine diagnostic reasoning.^21,30^ Frequency of discrepancy between the original and final diagnosis was not adjudicated, because the goal of the comparison process was reflection and not measurement. At completion of the track, residents reviewed both of their portfolios with the track director to identify patterns or biases in their diagnostic process. Blank depth and breadth portfolios are available in eAppendices 2 and 3.

Residents also dedicated 3–4 months to a scholarly project with a faculty mentor. Projects were resident-initiated and designed to foster a reflective approach to patient care and establish a scholarly foundation for a career in academic medicine.^18,19^ Some projects focused on principles of clinical mastery, such as creating a digital tool for a prediction-based approach to longitudinal clinical feedback. Others focused on patient care topics, such as the development of neurology patient education materials for non–English-speaking patients. Other projects focused on quality improvement and systems of care, such as the production of clinical guidelines for pediatric neurologic emergencies.

### Planned Analysis

We assessed our curriculum using a combination of methods. Achievement of our supporting curricular objectives (Table 1, column 2) was assessed through audits of patient portfolios, in-depth case analyses, and scholarly project completion. The goals of these audits were to ensure that curricular components (1) were complete and (2) contained the appropriate form of clinical data in each field (e.g., naming specific anatomic structures in the field for “localization”). The goal was not to scrutinize contents in a way that would supplant track participants' independent evaluation. For example, detailed content analysis of depth portfolios was not performed to mirror the realities that participants will face in independent practice, where such oversight is not available and all phases of SRL (planning, execution, and reflection) must be performed unsupervised.

Learner satisfaction was assessed using surveys on curricular elements. After completing the curriculum, participants completed an exit survey and then 3 subsequent annual surveys during fellowship or independent practice to track their perceptions of the curriculum. The exit survey contained 5-point Likert scales asking residents to rate the usefulness of all curricular elements of the track, including didactics and clinical rotations. Annual follow-up surveys asked residents to rate the usefulness of selected curricular elements, such as the didactic curriculum or the clinical rotations in aggregate (Supplementary Materials). Follow-up surveys also asked residents to indicate their primary career focus at that time. All surveys were piloted with members of an advisory council of faculty clinician-educators who assisted in designing the track; piloting the survey with residents was not feasible because the curriculum was hypothetical. Surveys were then formally administered to enrolled residents through email, and anonymous results were maintained in a secure encrypted online folder. Surveys are available in eAppendices 4–7.

### Research Ethics and Informed Consent

This 8-year study was approved by the University of California San Francisco Institutional Review Board (IRB Number 17-21488). All track members consented to participate in the study of the Master Clinician curriculum.

### Data Availability

Anonymized data not provided in the article and may be shared at the request of any qualified investigator for purposes of replicating procedures and results.

## Results

Five neurology residents completed the clinical mastery track curriculum from July 2017 to June 2022. Enrollment over this time frame was higher than in other non–research-based resident training tracks, including education (n = 2) and global health (n = 2), but was less than in clinical research (n = 14) and laboratory research (n = 24) tracks. Three residents have enrolled in the track between 2022 and 2026.

A convenience sample of 10 fourth-year residents who did not participate in the Master Clinician track were compared with track participants. This convenience sample of residents was drawn from the same postgraduate year as track residents. To investigate selection bias, we compared baseline metrics between these 2 groups. Track and nontrack residents had comparable milestone ratings in the spring before the academic year when starting the track (3.8 vs 3.7 of 5, SD 0.3, *p* = 0.55).

Master Clinician track residents rated didactics highly (4.6/5, n = 5 on a scale where 1 = not useful and 5 = very useful). Sessions on cognitive biases and teaching strategies received the highest ratings (5/5), followed by the master clinician panel and CME didactics (4.7/5). All other didactics scored 4 of 5 or higher except for the cost/benefit analysis session (Table 2). Outside the didactic curriculum, the independent project and neurology clinical rotations were the highest scoring elements of the track (both 5/5), followed by the breadth portfolio (4.8/5). In their responses to the open-ended question querying “specific components of the curriculum you found especially helpful,” participants emphasized their portfolios, neurologic clinical rotations, and multidisciplinary experiences related to their neurologic subspecialty. All curricular elements maintained high ratings over 3 years of postresidency follow-up, and all 5 residents maintained a clinically focused career for 2 years, whereas by year 3, several had moved toward other academic focuses (Table 3). Narrative comments by these residents indicated that they maintained a significant portion of clinical effort. None of the 5 residents had careers focused on quality improvement or global health by 3 years of follow-up.

**Table 2:** Exit Surveys of Track Residents (N = 5) Indicating Ratings of Curricular Elements in Descending Order (1 = Not Useful, 5 = Very Useful)

Curricular element	Average score	SD
Independent project	5	0
Neurology rotations	5	0
Breadth portfolio	4.8	0.4
Depth portfolio	4.4	0.8
Multidisciplinary rotations	4.4	0.8
Didactics	4.6	0.5
Cognitive bias, medical decision making	5	0
Teaching strategies	5	0
Master clinician inspirational panel	4.7	0.5
Continuing medical education	4.7	0.5
Adaptive expertise, clinical uncertainty	4.6	0.5
Communication strategies and conflict	4.6	0.5
Humanism, professional and physician identity	4.5	0.5
Cultural competency and diversity	4.4	0.5
Cost/benefit analysis	3.8	0.4

**Table 3:** Yearly Surveys of Track Residents (N = 5) Indicating Ratings of Master Clinician Track Curricular Elements (1 = Not Useful, 5 = Very Useful) Over 3 Years After Residency Graduation

Curricular element	Postresidency year 1 average score (SD)	Postresidency year 2 average score (SD)	Postresidency year 3 average score (SD)
Usefulness of Master Clinician curriculum	5 (0)	4.6 (0.4)	4.6 (0.5)
Didactics overall	4.8 (0.4)	4.3 (0.8)	4 (0.6)
Neurology rotations	4.6 (0.5)	5 (0)	4.8 (0.4)
Multidisciplinary rotations	4.2 (0.7)	4.8 (0.4)	5 (0)
Portfolios	4.2 (0.7)	4.5 (0.5)	4.8 (0.4)
Independent project	4.4 (0.5)	4.5 (0.9)	4.6 (0.5)
Primary career focus	Number of residents indicating field as their primary career focus		
Clinical neurology	5	5	2
Education	0	0	2
Clinical research	0	0	1

Breadth portfolios contained an average of 32 patients (range 10–63). The average self-assessed diagnostic accuracy was 70% (range 60%–82%). Participants provided an average of 1.3 primary diagnostic considerations per patient (range 1.2–1.3) and 2.7 alternative diagnoses (range 1.5–4.6). Breadth portfolio patients had an average of 1.2 final diagnoses (range 1–1.3) because participants determined that some patients had multiple concurrent neurologic processes.

Depth portfolios contained a total of 39 cases, averaging 8 patients per resident (range 7–9, Table 4). Audits demonstrated that residents completed portfolios for all included cases without any missing data. Depth portfolios of all 5 residents demonstrated that all track curricular topics were rated as either somewhat applicable or very applicable to at least 1 case. Depth portfolio cases had an average of 10 curricular topics rated as somewhat or very applicable. Topics most frequently rated as “very applicable” were clinical uncertainty, communication strategies, and cognitive bias. Topics most often rated as “somewhat applicable” were adaptive expertise, professional identity, quality of care, and medical systems. Conflict and cost/benefit analysis were most commonly rated as “not applicable.”

**Table 4:** Number of Depth Portfolio Cases Rated by Track Residents to Be Very Applicable, Somewhat Applicable, or Not Applicable to Each Curricular Topic (Total Depth Portfolio Cases, N = 39)

Topic	Number of cases rating topic as not applicable (%)	Number of cases rating topic as somewhat applicable (%)	Number of cases rating topic as very applicable (%)
Adaptive expertise	2 (5)	22 (56)	15 (39)
Clinical uncertainty	2 (5)	13 (33)	24 (62)
Cognitive bias	3 (8)	15 (39)	21 (54)
Communication strategies	5 (13)	12 (30)	22 (56)
Conflict	23 (59)	10 (26)	6 (15)
Cost/benefit analysis and high-value care	19 (49)	15 (39)	5 (13)
Cultural competency and diversity	11 (28)	16 (41)	12 (31)
Deliberate practice	3 (8)	31 (80)	5 (13)
Humanism	15 (39)	12 (31)	12 (31)
Medical systems	4 (10)	20 (51)	15 (39)
Multidisciplinary care	6 (15)	17 (44)	16 (41)
Professional and physician identity	8 (21)	22 (56)	9 (23)
Quality of care	7 (18)	21 (54)	11 (28)

Depth portfolio narrative comments supported resident ratings of topic applicability. One participant describing a case in which conflict was rated “highly applicable” wrote, “Consulting teams were being candid about most likely dx and dismal prognosis before our team had spoken to family about the specifics.” Another participant describing a case rated “highly applicable” to topics of clinical uncertainty and cognitive bias wrote, “Anchoring was made early on neuro-Sweet syndrome given dermatology's initial confidence in this diagnosis, but ultimately once Acanthamoeba was identified elsewhere, her (low-dose) steroids were quickly stopped, and her response to infectious treatment further supported the diagnosis.”

All residents presented their scholarly projects as posters at an annual department research event. One resident also presented their project at a departmental education retreat and, now as faculty, continues to disseminate a clinical reasoning tool from that project to residents each year.

## Discussion

Neurology training programs cannot create clinical experts in 4 years, but they can introduce graduates to the skills and experiences to reach that goal. The intermediate goal is the development of self-regulated learners who seek challenges that exceed standard training requirements, habitually monitor their performance, and reflect on their practice to adjust future approaches. To develop the habit of seeking new and challenging environments and topics, Master Clinician track residents enrolled in multidisciplinary rotations. To foster the lifelong habit of patient tracking and reflection, residents maintained patient case portfolios. To solidify the examination of their practice, residents examined outcomes in their portfolios and engaged in learning-focused neurology rotations. Case portfolio audits demonstrated that residents successfully applied all curricular topics to clinical scenarios at some point during the track. Finally, to facilitate a future career in academics and support a foundation in inquiry aimed toward advancement of medical knowledge, residents performed a scholarly project. Through these combined strategies, we aimed to instill the MAL framework as a foundational habit of expert practice. The components of the track were favorably rated by its members, and these high ratings endured over 3 years of follow-up.

Our experience provides insights for other program leaders. First, master clinician training programs can be structured to include didactics, clinical experiences, and independent projects. Didactics should be clinically relevant, promote resident engagement, and be scheduled during protected time when possible. Furthermore, because residency programs already cover clinical neurology extensively, didactics should be focused on metacognitive aspects of learning to promote a MAL orientation. Furthermore, learning-focused clinical experiences provide residents with time and opportunity to apply lessons learned from didactics. Multidisciplinary clinical experiences should be framed to residents as valuable experiences that will not be readily available to them after graduation. When soliciting faculty members to deliver track didactics, we found that making a request that highlighted their reputation for clinical prowess was persuasive. Drawing on a diverse range of faculty members, including non-neurologists, broadens resident exposure to different perspectives and distributes the curricular workload to promote sustainability. Finally, because the principles of the MAL are agnostic to specialty, our program could be adapted for residency programs in other areas of medicine as well. It would be possible for different specialty programs at the same institution to pool resources and create a master clinician track accessible to residents across multiple programs. Such collaboration would significantly augment sustainability.

This curriculum was implemented at a single neurology residency program, which may limit its generalizability. Track participants self-selected into the program, which may introduce bias. The small number of track participants and the associated sample size precluded statistical conclusions regarding comparative or temporal data. Our study also did not evaluate potential effects on professional identity formation or career advancement. Following a larger cohort of residents over a longer period would allow for a more detailed characterization of career trajectories, and qualitative analysis could be used to determine the extent to which those trajectories were influenced by participation in the Master Clinician track. Finally, residents only completed portfolios at the end of the curriculum, and portfolio assessments relied on resident self-reporting and faculty review without rubric-based evaluation or external validation. This may limit the objectivity and reproducibility of conclusions drawn from portfolio content.

Participants in the Master Clinician track provided suggestions in their exit and follow-up surveys that have guided curricular improvements. One suggestion was to create a repository of teaching cases for resident review. Another was to create a library of key publications on master clinician development for participants to reference from. Finally, multiple residents requested increasing mentorship throughout the track, specifically requesting multiple check-in points with track leadership to review portfolios, discuss complex cases, and contemplate clinical skills development.

We successfully developed a 6-month track to introduce neurology PGY-4 residents to the skills and foundational experiences to become academic neurologists with a primary career focus on clinical excellence. This track was highly rated by the participants and alumni. Neurology programs seeking to promote career pathways in clinical excellence should consider using a combination of customized clinical experiences, small group didactics, scholarly projects, and patient portfolios to promote self-directed longitudinal skill development tailored to an academic career.

## Glossary

MAL: Master Adaptive Learner
SRL: self-regulated learning
